# Dietary Patterns of Off-Reserve Indigenous Peoples in Canada and Their Association with Chronic Conditions

**DOI:** 10.3390/nu15061485

**Published:** 2023-03-20

**Authors:** Pardis Keshavarz, Ginny Lane, Punam Pahwa, Jessica Lieffers, Mojtaba Shafiee, Kelly Finkas, Marisa Desmarais, Hassan Vatanparast

**Affiliations:** 1 College of Pharmacy and Nutrition, University of Saskatchewan, Saskatoon, SK S7N 5E5, Canada; 2 Margaret Ritchie School of Family and Consumer Sciences, University of Idaho, Moscow, ID 83844, USA; 3 Department of Community Health and Epidemiology, College of Medicine, University of Saskatchewan, Saskatoon, SK S0G 5L0, Canada; 4 Health and Social Development, Cowessess First Nation, Cowessess, SK S0G 5L0, Canada; 5 Health and Social Development Department, Community Dietitian, Cowessess First Nation, Cowessess, SK S0G 5L0, Canada; 6 School of Public Health, University of Saskatchewan, Saskatoon, SK S7N 5E5, Canada

**Keywords:** dietary patterns, cluster analysis, Indigenous ethnic group, Canadians, chronic diseases

## Abstract

Nationally representative nutrition surveys (Canadian Community Health Survey (CCHS) Cycle 2.2, Nutrition 2004 and 2015) were used to examine dietary patterns and their association with socioeconomic/sociodemographic factors and chronic conditions in off-reserve Indigenous population in Canada. A cluster analysis was used to identify dietary patterns (DPs), and the Nutrient Rich Food Index (NRF 9.3) was used as the diet quality score and stratified by age/gender groups. In 2004 (*n* = 1528), the dominant DPs among Indigenous adults (age = 41 ± 2.3) were “Mixed” (mean NRF = 450 ± 12) and “Unhealthy” among men (mean NRF = 426 ± 18), “Fruits” among women (mean NRF = 526 ± 29), and “High-Fat/High-Sugar” among children (age = 10.2 ± 0.5) (mean NRF = 457 ± 12). In 2015 (*n* = 950), the dominant DPs were “Unhealthy” (mean NRF = 466 ± 6), “Mixed” (mean NRF = 485 ± 21), Healthy-Like (mean NRF = 568 ± 37), and “Mixed” (mean NRF = 510 ± 9) among adults (age = 45.6 ± 2.2), men, women, and children (age = 10.9 ± 0.3) respectively. The majority of Indigenous peoples had the “Unhealthy” DP with a low diet quality, which may contribute to a high prevalence of obesity and chronic diseases. The income level and smoking status among adults and physical inactivity among children were recognized as important factors that may be associated with the dietary intake of off-reserve Indigenous population.

## 1. Introduction

The prevalence of non-communicable diseases (NCDs) is increasing rapidly, particularly among Indigenous populations in Canada [1]. Chronic heart diseases, diabetes, and high blood pressure have been shown to be more prevalent among Indigenous peoples than in their non-Indigenous counterparts [2]. Although genetic susceptibility may predispose Indigenous peoples to an increased risk of chronic diseases [3], other factors such as metabolic status [4], lifestyle [5], diet, trauma, and stress [6] appear to mediate the risk of disease.

Over the past century, a dramatic shift in lifestyle has occurred among Canadian Indigenous populations from one that involves a high level of physical activity such as hunting and gathering to one that involves less movement and is more inactive [7,8]. Furthermore, a nutrition transition has also taken place among these communities that is characterized by the substitution of traditional food resources (i.e., a diet rich in fish, wild meats, berries, and roots) with foods high in refined ultra-processed carbohydrates and fat and low in fiber [9]. In addition, other factors such as low income, food insecurity, and racism, which cause trauma and stress [6], have resulted in negative changes in lifestyle and dietary patterns (to an unhealthy dietary intake) by Indigenous communities [10,11]. Indigenous communities often rely on traditional food systems that are based on hunting, fishing, and gathering. However, these traditional food systems have been disrupted by factors such as environmental degradation, loss of land and water resources, and the introduction of non-native species that compete with or prey upon traditional species [12]. Food insecurity has significant negative effects on the health and well-being of Indigenous people, which can lead to chronic health conditions [13,14].

The nutrition transition along with a sedentary lifestyle has resulted from an increase in the prevalence of obesity-related diseases in many different populations [15]. In a Canadian study, Indigenous people experienced the greatest the risk of developing hypertension with increased body mass index (BMI) or waist circumference compared to other ethnic groups [16]. Another study among an Indigenous population on a reserve in northwestern Ontario showed that adult women were more likely to have a higher BMI and a much higher percentage body fat compared to men [9]. The causes of obesity are complex and multifaceted and encompass factors such as dietary intake, socioeconomic status, sociodemographic characteristics, and lifestyle. The complexities of various diets can be understood through dietary pattern analysis, which sets it apart from the traditional single-nutrient focus approach and may inform a comprehensive approach to disease prevention or treatment [17].

Dietary patterns have been shown to vary across genders [18]. The relationship between dietary patterns and chronic diseases is influenced by gender. From a biological perspective, women are at lower risk for developing cardiovascular diseases (CVDs) compared to men [19], although obesity is more prevalent among women [20], and women are at higher risk for osteoporosis [21]. From a dietary intake perspective, women are more likely to be conscious of making healthy choices and are more likely to follow dietary health advice than men [22].

The Canadian Community Health Survey (CCHS) 2004 and 2015 nutrition data provide the opportunities to address the gap in knowledge regarding dietary patterns of Indigenous peoples and their association with chronic diseases and to examine trends over time. To the best of our knowledge, this study was the first to comprehensively examine the dietary patterns of off-reserve Indigenous peoples in Canada using CCHS data and a gender perspective. The aim of this study was to identify the dietary patterns of off-reserve Indigenous men, women, and children in Canada and their association with chronic conditions in 2004 and 2015 while considering related sociodemographic and socioeconomic conditions.

## 2. Materials and Methods

### 2.1. Study Population

Our analysis used information obtained from two cross-sectional surveys: the CCHS Cycle 2.2, Nutrition (2004) and the CCHS 2015. The CCHS 2015 is the most comprehensive and up-to-date survey on the dietary intake of Canadians. These surveys encompass the population of Canada aged one year and above living in the 10 provinces except for individuals residing on reserves and other Indigenous settlements, full-time members of the Canadian Forces, and the institutionalized population. The survey includes self-reported information on selected health conditions, a 24 h dietary recall, height and weight (measured), and socioeconomic and demographic characteristics [23,24]. The overall sample size was 35,000 in 2004 and 20,487 in 2015. For this study, we excluded the non-Indigenous population, pregnant and lactating women, individuals younger than 4 years of age, and individuals with invalid self-reported dietary recalls (as defined by Statistics Canada). This cross-sectional study included 1528 and 950 individuals who self-identified as Indigenous aged 4–71 or older in 2004 and 2015, respectively. Indigenous included all participants who self-reported single or mixed Indigenous ancestry.

### 2.2. Dietary Assessment

Comprehensive information on dietary intake was obtained through the modified five-step 24 h recall method following the Automated Multiple Pass Method (AMPM) developed by the U.S. Department of Agriculture (USDA, Washington, DC, USA) [23,25,26]. Participants were asked to recall all beverages and foods consumed in the previous 24 h [27].

In the early 1990s, the Bureau of Nutritional Sciences (BNS, Ottawa, ON, Canada) at Health Canada developed the “BNS food and recipe groups”. This system of food grouping consists of two classifications: one for basic foods and another for recipes, which are helpful in evaluating the consumption of various food items.

To assess diet quality, we used the modified nutrient-rich food (NRF) index. This index is a useful tool because it considers nutrients that are crucial for public health, such as proteins, fibers, vitamin A, vitamin C, vitamin D (replacing vitamin E in the original NRF index), calcium, iron, magnesium, and potassium. It also includes nutrients that should be limited, such as fat, added sugars, and sodium [28]. The NRF 9.3 score, which is based on a daily nutrient intake normalized to 2000 kcal of energy intake and expressed as percentages of national daily values (DVs), was used in our study. Canada has a DV for total sugars instead of added sugars. The Canadian Nutrient File (CNF) does not list added sugars, hence total daily sugar consumed was used as a nutrient to limit.

### 2.3. Sociodemographic and Lifestyle Characteristics

Sociodemographic and lifestyle factors including age, gender, income, education, physical activity, smoking, and food security, as well as BMI calculated from measured height and weight, were divided into categories. Age was grouped into three categories: children (4 years or older to less than 18 years old), young adults (18 years or older to less than 41 years old), and adults (41 years or older). CCHS includes annual household income based on the number of household members. Income was split into two categories (merging 10 deciles), with low income defined as deciles 1 to 5 and high income defined as deciles 6 to 10. Education (household) was categorized as below bachelor’s level or at or above bachelor’s level. Physical activity for adults was divided into two categories: those who met the physical activity guideline of 150 min of moderate to vigorous physical activity per week (according to Canadian physical activity guidelines and WHO recommendations) and those who did not. Sufficient physical activity for children aged 6 to 17 was considered to be 60 min of moderate to vigorous physical activity per day in the past 7 days with categories defined as meeting or not meeting this level. Smoking status in the CCHS was divided into two main categories: smokers and non-smokers (with former smokers classified as smokers if they stopped smoking less than a year prior to the interview). BMI for adults was based on WHO classification with categories for normal weight (BMI 18.5–24.9), overweight (BMI 25–29.9), and obese (BMI 30 or above) and included categories for obese I (BMI 30–34.9), obese II (BMI 35–39.9), and obese III (BMI above 40). Participants classified as underweight (BMI less than 18.5) were excluded from the analysis. BMI Z-scores for children under 18 years old were calculated using the WHO AnthroPlus software [29].

The categorization of household food security was divided into two groups: food secure and food insecure, which encompassed marginal, moderate, and severe food insecurity. The results were interpreted regionally based on five regions in Canada: Atlantic (Newfoundland and Labrador, Nova Scotia, New Brunswick, and Prince Edward Island), Ontario, Quebec, Prairies (Manitoba, Saskatchewan, and Alberta), and British Columbia.

The CCHS survey comprised self-reported data on several chronic health conditions that included cancer, diabetes, high blood pressure, cardiovascular disease, and osteoporosis. Adult respondents were asked if they have been diagnosed with a chronic disease by a healthcare professional and if the diagnosis has lasted for at least 6 months. Obesity (obese I, obese II, and obese III) was determined based on measured height and weight.

### 2.4. Statistical Analysis

All statistical analyses were performed using SAS software (version 9.3; SAS Institute Inc., North Carolina, NC, USA). To determine population parameters such as coefficients of variation, confidence intervals, and standard errors, we used the bootstrap balanced repeated replication method with 500 repeats. The survey weights provided in the master files were used for all individuals to ensure that the sample remained nationally representative. Results with a *p*-value ≤ 0.05 were considered statistically significant.

### 2.5. Cluster and Regression Analyses

The BNS food groups were consolidated from 51 groups into 37 groups based on similarities. The intake (in grams) from each of the 37 food groups was calculated separately for each individual using their first 24 h recall. The contribution of each food group to the total gram intake was used for conducting the cluster analysis. A k-mean cluster analysis was used to identify the dietary patterns among Indigenous peoples from CCHS 2015 and 2004 data for children and adults separately. The clustering procedure began with k random centers, after which the observations were allocated to the closest centroid. The crucial aspect of the clustering process was establishing the number of clusters (k). In the case of dietary patterns, determining the number of clusters is typically difficult due to the lack of theoretical background information. Therefore, two approaches were employed to determine the number of clusters (k): (1) examining the kink in the screen plots of within sum of squares (WSS), logarithm of WSS, η2 coefficient, and proportional reduction of error (PRE) coefficient [30]; and (2) by using the “cluster stop” command. To mitigate the sensitivity of the cluster analysis to outliers, a box plot was created for each variable to identify and remove outliers.

After running the cluster analysis, names were assigned to each cluster based on the dominant food groups’ contributions (calculated as the sum of the mean intake of food groups divided by the mean intake of each food group) and NRF. This process was also completed separately for men and women. Then, the sociodemographic and lifestyle characteristics were compared across different clusters (separately for adults, men, women, and children).

A logistic regression analysis (PROC LOGISTIC) was used to evaluate the link between dietary patterns and health outcomes. The surveys collected data on individuals aged 30 years and above with chronic diseases, who were defined as those with at least one diagnosed chronic condition lasting six months or more (including diabetes, high blood pressure, cardiovascular diseases, osteoporosis, and cancer). The first step involved using bivariate logistic regression to evaluate the association between potential confounders or interaction terms such as age, income, education, smoking status and BMI. This was followed by multivariable logistic regression to investigate the association between chronic conditions (i.e., obesity and chronic diseases) and dietary patterns.

## 3. Results

The cluster analysis of Indigenous adults’ dietary habits in 2004 rendered three dietary patterns: “Soups”, “Fruits/Vegetables/Pasta”, and “Mixed”. In 2015, two dietary patterns were identified: “Unhealthy” and “Mixed”. The dietary patterns varied according to gender. In 2004, men had three dietary patterns (“Unhealthy”, “Soups”, and “Potato”), while women had four (“Soups”, “Fruits”, “Mixed”, and “Vegetables”). However, in 2015, women had two dietary patterns (“Mixed” and “Healthy-like”) and men had three (“Mixed”, “Soups”, and “High-Fat”). The names of the clusters were assigned based on the food group that dominated in each cluster.

Table 1 presents the dietary patterns of Indigenous adults along with their sociodemographic and lifestyle characteristics. The top five food groups in each cluster are listed as ranked by their contribution to the total daily intake (g).

Among Indigenous adults in 2004 (Table 1), the most common dietary pattern was “Mixed”, and the “Fruits and Vegetables/Pasta” pattern had the highest diet quality score. However, in 2015, the “Unhealthy” dietary pattern was more prevalent among Indigenous adults (weighted *N* = 289,433), and the “Mixed” dietary pattern had a significantly higher diet quality score. The proportion of households with higher income was significantly higher in the “Fruits and Vegetables/Pasta” 2004 dietary pattern compared to other clusters.

The dietary patterns specific to men and women are displayed in Table 2 and Table 3, respectively, for the years 2004 and 2015.

Most Indigenous men had the “Unhealthy” dietary pattern in 2004 and “Mixed” dietary pattern in 2015 (Table 2). The highest diet quality score was seen among men with the “Potato” dietary pattern in 2004 and the “Mixed” dietary pattern in 2015. The “Soups” and “Unhealthy/High-Fat” dietary patterns were observed in both years.

In both 2004 and 2015, Indigenous women had healthier dietary patterns compared to men (Table 3). The majority of women consumed the “Fruits” dietary pattern in 2004 and the “Healthy-like” in 2015. Indigenous women who followed the “Vegetables” and “Healthy-like” dietary patterns had the highest diet quality scores in 2004 and 2015, respectively.

The dietary patterns of Indigenous children in both 2004 and 2015 are presented in Table 4.

Three dietary patterns were identified for Indigenous children in 2004 (Table 4): “Soups”, “High Fat-High Sugar”, and “Mixed”. All individuals in the “Soups” dietary pattern were physically active. In 2015, two dietary patterns were identified (“Mixed” and “Unhealthy” dietary patterns) among Indigenous children, and the NRF score for the “Mixed” dietary pattern was significantly higher than the “Unhealthy” cluster. Those with the “Unhealthy” dietary pattern had the highest percentage of physical inactivity. The “High Fat-High Sugar” and “Mixed” dietary patterns were the most common in 2004 and 2015, respectively.

### 3.1. Prevalence of Obesity and Chronic Diseases across Dietary Patterns

**Figure 1:**
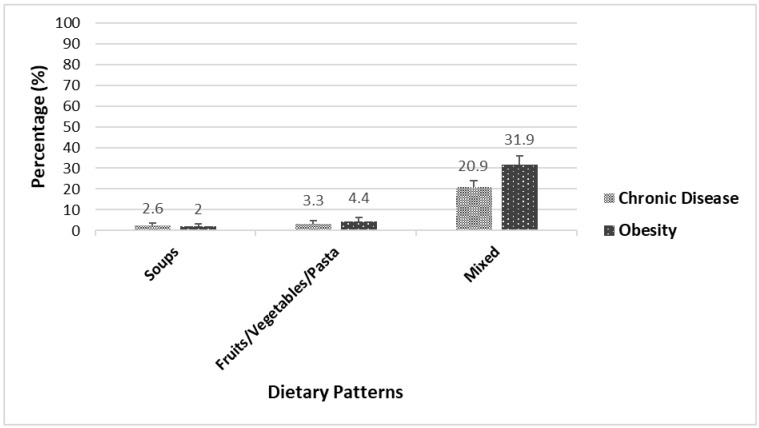
Prevalence of chronic diseases and obesity across dietary patterns among Indigenous adults in 2004.

**Figure 2:**
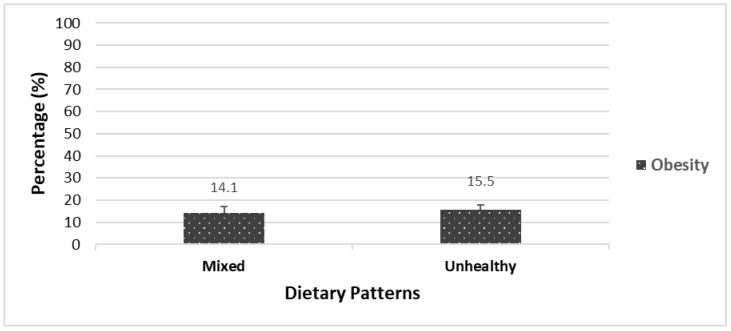
Prevalence of obesity across dietary patterns among Indigenous adults in 2015.

Figure 1 and Figure 2 illustrate the prevalence of chronic diseases and obesity among Indigenous adults in 2004 and 2015, respectively, based on different dietary patterns.

In 2004, the prevalence of chronic diseases (21%) and obesity (32%) was significantly higher among Indigenous adults following the “Mixed” dietary pattern compared to those following the “Soups” and “Fruits/Vegetables/Pasta” dietary patterns (Figure 1).

In 2015, Indigenous adults following the “Unhealthy” dietary pattern had a higher prevalence of obesity (14%) compared to those with the “Mixed” dietary pattern; however, this difference was not statistically significant (Figure 2). The prevalence of chronic diseases across dietary patterns in 2015 was not available due to the small sample sizes.

### 3.2. Association of Related Factors across Dietary Patterns

A regression analysis was carried out on three groups: all adults, men, and women. The statistical analysis of chronic diseases was based on adults aged 30 years or older, while for obesity it was based on adults aged 18 years or older. The significant associated factors (*p* < 0.05) are presented in Table 5.

In 2004 (Table 5), Indigenous adults with high income were 0.14 less likely to have a chronic disease. In 2015 (Table 5), Indigenous individuals who were over 45 years of age were more likely to have a chronic disease. There were no significant associations between the related variables and obesity among Indigenous adults in 2004 and 2015.

In 2015, Indigenous men ≥45 and >65 years of age (OR: 4; 95% CI = 2.5–5) and 65 years old and over (OR: 7; 95% CI = 3–7.8) were significantly more likely to have chronic diseases. There was no other significant association between the related variables and chronic diseases among Indigenous women.

In 2004, there were no statistically significant associations between the selected factors and obesity among Indigenous men. In 2015, Indigenous men with high income (OR: 5.5; 95% CI = 1.7–7.5) and those who were physically inactive (OR: 4.2; 95% CI = 1.1–6.3) were more likely to be obese. No significant associations were found between the related variables and obesity among Indigenous women in both years except that physically inactive Indigenous women were four times more likely to be obese compared to active women in 2004 (OR: 4.2; 95% CI = 1.4–6.2).

## 4. Discussion

### 4.1. Dietary Patterns and Their Associated Factors among Indigenous Adults

Indigenous people are recognized as having distinctive social, cultural, and health needs within the mainstream societies where they live. This was the first study in Canada to investigate the dietary patterns of the Indigenous population and their association with chronic diseases, obesity, and related factors using a nationally representative sample. Our results demonstrated that there were three dietary patterns among Indigenous adults in 2004: “Mixed”, “Fruits/Vegetables/Pasta”, and “Soups”; while in 2015, two dietary patterns were identified: “Mixed” and “Unhealthy”. In both 2004 and 2015, the “Mixed” and “Unhealthy” dietary patterns were the most common among the population. The diet quality scores varied considerably between dietary patterns in both years. The “Fruits/Vegetables/Pasta” and “Mixed” dietary patterns had the highest diet quality scores among Indigenous adults in 2004 and 2015, respectively.

Other research found that the “Sandwich” category, which included sandwiches, pizza, submarines, hamburgers, and hotdogs, was a popular dietary pattern among Indigenous adults [31,32]. Johnson-Down et al. (2014) found three dietary patterns: “Inland” (with greater loadings on fish), “Coastal” (more game and freshwater fish), and “Junk food” among adult participants from seven communities in northern Québec; the consumption of “Junk food” was found to be common [33]. The traditional food intake among the Inuit in Nunavut was associated with higher diet quality especially with regard to protein intake and a number of minerals and vitamins [34]. Indigenous people in the Canadian Arctic who consumed no traditional foods had a lower diet quality score and a higher consumption of carbohydrates, fat, and sucrose [35]. It appears that consuming the traditional diet is linked to a higher diet quality score in this group. The decrease in traditional food consumption is due to the nutrition transition leading to increased consumption of market foods, which often contain ultra-processed foods [8,35]. Economic barriers, climate change, having an active hunter in the household, and changing food preferences have impacted traditional food consumption in Indigenous communities [36,37,38].

In our study, we found that the “Soups” dietary pattern was present among both adult men and women in both 2004 and 2015. In 2004, the “Unhealthy” and “Mixed” dietary patterns were most common among men, while in 2015 the “Mixed” dietary pattern was the most prevalent. On the other hand, women in both 2004 and 2015 were found to have a higher proportion following the “Fruits” and “Healthy-like” dietary patterns. Our findings showed that Indigenous women were more likely to follow a healthier diet compared to men. However, Indigenous heath reports in 2008 indicated that Indigenous women (living off-reserve) had a higher intake of snacks, sodium, and carbohydrates compared to non-Indigenous women [32]. This report also indicated Indigenous men consumed more grain products, vegetables, fruits, and meat compared to women.

Our findings showed that in 2004, Indigenous adults who followed the “Soups” dietary pattern had lower income, while those who followed the “Fruits/Vegetables/Pasta” dietary pattern had higher income. In 2015, those who followed the “Unhealthy” dietary pattern included a higher percentage of smokers compared to those who followed the “Mixed” dietary pattern. These results were in agreement with the Indigenous health report from 2008, which stated that Indigenous people in lower-income households were more likely to consume unhealthy food [32]. Our findings suggested that income level and smoking status may be related to the dietary patterns of Indigenous adults.

In 2004, higher income was negatively associated with chronic disease, while in 2015 older age was positively associated with chronic disease. Furthermore, being inactive was also positively associated with obesity among Indigenous men and women in both years. Setiono et al. (2019) reported that following the “meat and fried foods” and “processed foods” dietary patterns was positively associated with obesity and diabetes in six American Indian communities [39]. Another analysis using CCHS 2004 Cycle 2.2 data indicated higher rates of obesity among Indigenous adults in Canada compared to non-Indigenous individuals [32]. Living in low-income households was also associated with a higher rate of obesity among Indigenous peoples [32]. Since income level likely captures the resources an individual has to engage in certain lifestyle behaviors, having a low income may limit an individual’s capacity to invest resources in physical activity or healthy foods. However, the analysis was cross-sectional, making it unclear whether obesity influences income or vice versa [40]. Sarkar et al. (2019), suggested that restoration of native ecosystems, reviving traditional food preparation knowledge and traditional food crop cultivation, and promoting the consumption of traditional plant-based foods are important steps in building dietary support strategies against NCDs in Indigenous communities [41].

### 4.2. Dietary Patterns and Their Associated Factors among Indigenous Children

In 2004, three dietary patterns were identified among Indigenous children: “Soups”, “High-Fat/High-Sugar,” and “Mixed”. In 2015, Indigenous children had dietary patterns similar to adults that included “Mixed” and “Unhealthy.” The majority of children had the “High-Fat/High/Sugar” and “Mixed” dietary patterns. These results were consistent with a previous study that reported a shift from traditional foods to a Westernized diet among Australian Indigenous children and adolescents (including increased consumption of fried foods and confectionaries) [42]. According to the results of Statistics Canada’s 2006 Aboriginal children’s survey (ACS), about two-thirds of all Indigenous children living off-reserve consumed processed foods and fast food at least once a week, and more than fifty percent had sweets, salty snacks, and desserts at least once a day [43]. This shift toward unhealthy dietary intake among Indigenous children could be related to a sedentary lifestyle as well as limited accessibility to and availability of high-quality foods such as fresh fruits and vegetables. On the other hand, the increased availability of store-bought foods that are appealing to children may decrease their overall diet quality [43].

Physical activity appeared to be an important factor for Indigenous children in both years. Those with “High-Fat/High-Sugar” and “Unhealthy” dietary patterns were significantly more likely to have low physical activity levels. Foulds et al. (2011) also reported that a large proportion of the British Columbian Aboriginal population aged 16 or older were physically inactive [44]. There is no further information available in the literature regarding the dietary patterns and physical activity levels of Canadian Indigenous children.

The dietary intake of the majority of the Indigenous population in Canada moved toward unhealthy and processed foods from 2004 to 2015, which raises concerns regarding the consequent higher prevalence of chronic diseases and obesity in this population (specifically among children). Promoting the revival of Indigenous food systems and the consumption of traditional and healthy foods can have positive impacts on health outcomes and cultural heritage preservation.

Approaches such as local community engagement in culturally appropriate health promotions initiatives aimed at promoting healthy eating (with inclusion of traditional food), wellness, and physical activity programs in schools are also recommended to raise public awareness and promote healthy lifestyles. We suggest that future studies focus on conducting a cohort studies among Indigenous populations to investigate social, cultural, and environmental constraints affecting dietary patterns in association with health and chronic diseases. It is crucial to fill the knowledge gaps in developing culturally based gender/age-specific nutrition information aligned with Canada’s Food Guide to inform healthy food choices among the Indigenous population. Addressing these knowledge gaps will enhance the capacity of our public health system to support Indigenous population to achieve optimal health and well-being. Moreover, systematic change likely will not occur by following a cookie-cutter approach based on a single recommendation, publication, or policy while not considering the elements of culture and local community-specific circumstances. Community-based, comprehensive, well-rounded recommendations and policies that are frequently repeated, culturally adopted by the communities, and socially popular are needed to support healthy food choices among Indigenous population.

Our study on the dietary patterns of Indigenous Canadians with a focus on gender differences, socioeconomic status, sociodemographic factors, lifestyle, and health outcomes fills a significant knowledge gap. First, we used a combination of a priori (diet quality score) and a posteriori (cluster analysis) approaches in this study to assess diet quality and explore the intercorrelation of food groups and distinct patterns of food intake based on a population-driven approach. Secondly, the cluster analysis provided a superior method for assessing dietary patterns compared to the single nutrient method using a population-driven approach that identified clusters of individuals within a population who shared similar characteristics [45]. Third, using data from the CCHS Cycle 2.2, Nutrition (2004) and CCHS 2015 provided national population data that is generalizable to the Indigenous population in Canada and allows for examination of dietary pattern trends over time. However, there are a few limitations to consider such as the use of cross-sectional data that could not determine cause-and-effect relationships. Secondly, dietary patterns were identified based on the first 24 h dietary recall, which may not have accurately reflected an individual’s usual diet and may have resulted in potential over- or under-reporting. Third, there is no established standard for determining the number of clusters, which required a subjective decision to be made based on existing information. In this study, as outlined in the Materials and Methods section, we used both cluster stop and box plot approaches to mitigate this limitation.

## 5. Conclusions

The findings of this research provide researchers and policy makers with a comprehensive overview of the dietary patterns of off-reserve Indigenous peoples in Canada in 2004 and 2015. The majority of Indigenous people had an “Unhealthy” dietary pattern with a low diet quality score, which could lead to the development of obesity and chronic diseases. In addition, women had healthier dietary habits than men in both years. Among children, the dominant dietary pattern was also “Unhealthy,” which indicated a potential risk for chronic diseases later in adulthood. The significant factors associated with dietary patterns of the Indigenous population were income and smoking status among adults and physical inactivity among children.

## Figures and Tables

**Table 1:** Dietary patterns of Indigenous adults in 2004 and 2015.

		CCHS 2004									CCHS 2015					
		Adults									Adults					
**Dietary pattern**		Soups Wgt *N* ^a^ = 89,702			Fruits and Vegetables/Pasta Wgt *N* = 110,945			Mixed Wgt *N* = 270,138			Mixed Wgt *N* = 98,071			Unhealthy Wgt *N* = 289,433		
**Top five foods**		Food name	Mean ^b^ ± SD (g)	% ^c^	Food name	Mean ± SD (g)	%	Food name	Mean ± SD (g)	%	Food name	Mean ± SD (g)	%	Food name	Mean ± SD (g)	%
		1. Soups	538 ± 3.5	41	Vegetables	446 ± 30.1	31	Vegetables	78 ± 3.5	11	Vegetables	191 ± 16.8	13	Fast foods	171 ± 6.9	13
		2. Vegetables	95 ± 15	7	Fruits	187 ± 36.3	13	Fruits	59 ± 4.7	8	Fruits	168 ± 15.9	12	Vegetables	144 ± 7	11
		3. Potato	66 ± 1.4	5	Pasta	172 ± 30.1	12	Potato	56 ± 5	8	Fast foods	156 ± 18	11	Fruits	121 ± 9.2	9
		4. Beef	61 ± 22.3	5	Added fats	72 ± 7.7	5	Beef	52 ± 3.8	8	Whole grains	104 ± 10.6	7	Added fats	94 ± 8.6	7
		5. Whole grains	53 ± 13.2	4	Beef	61 ± 10	4	Added fats	51 ± 2.9	7	Soups	101 ± 20.7	7	Sweet baked goods	79 ± 5.9	6
NRF ^d^ (mean ± SD)		395.9 ± 17.8 *			**518.5 ± 24.7**			**449.7 ± 12**			**547.5 ± 4.2**			**466.3 ± 6.2**		
Age (mean ± SD)		42.8 ± 2.3			38.1 ± 2.6			42 ± 1.1			45.7 ± 2.2			45.5 ± 2.4		
Weight (kg)		83.7 ± 9.1			79.4 ± 6.2			78.5 ± 2.2			77.2 ± 0.9			86.9 ± 2.4		
Gender (% woman)		39.6			53.4			58.0			48.0			40.0		
BMI (%)	Normal	51.0			20.6			30.3			26.0			17.0		
	Overweight	7.8			36.7			31.8			20.0			38.5		
	Obese	41.2			42.7			37.8			54.0			44.5		
Income (%)	Low	**87.6**			**43.0**			**69.1**			52.0			46.0		
	High	**12.4**			**57.0**			**30.9**			48.0			54.0		
Education (%)	Below bachelor’s level	97.8			83.7			90.9			81.0			79.5		
	Bachelor’s level and above	2.2			16.3			9.1			19.0			41.0		
Smoking (%)	Non-smoker	25.4			57.7			47.7			**73.0**			**59.0**		
	Smoker	74.6			42.3			52.3			**27.0**			**41.0**		
Activity (%)	Active	29.2			48.3			38.9			52.0			46.0		
	Inactive	70.8			51.7			61.1			48.0			54.0		
Food security (%)	Food insecure	41.1			23.5			36.5			25.0			35.0		
	Food secure	58.9			76.5			63.5			75.0			65.0		

^a^ Weighted sample size; ^b^ values are mean (g) ± standard error of the mean (SEM); ^c^ percentage of contribution of the food group; ^d^ nutrient-rich food; * significant values are bolded (*p* < 0.05).

**Table 2:** Dietary patterns of Indigenous men in 2004 and 2015.

	CCHS 2004									CCHS 2015								
	Men									Men								
**Dietary pattern**	Unhealthy Wgt *N* ^a^ = 200,190			Soup Wgt *N* = 25,126			Potato Wgt *N* = 87,919			Mixed Wgt *N* = 140,819			Soup Wgt *N* = 40,828			High-Fat Wgt *N* = 63,532		
**Top five foods**	Food name	Mean ^b^ ± SD (g)	% ^c^	Food name	Mean ± SD (g)	%	Food name	Mean ± SD (g)	%	Food name	Mean ± SD (g)	%	Food name	Mean ± SD (g)	%	Food name	Mean ± SD (g)	%
	Vegetables	106 ± 10.4	14	Soups	662 ± 69	38	Potato	318 ± 30	25	Vegetables	138 ± 13	11	Soups	752 ± 87	37	Fast foods	625 ± 30	33
	Fruits	68 ± 9.5	9	Vegetables	247 ± 69	14	Vegetables	173 ± 23.6	14	Fruits	96 ± 11	8	Vegetables	209 ± 44	10	Vegetables	148 ± 22	8
	Beef	59 ± 6.5	8	Whole grains	90 ± 24.7	5	Beef	138 ± 22.9	11	Fast foods	95 ± 8	8	Whole grains	144 ± 44	7	Whole grains	139 ± 20	7
	Added fats	53 ± 4.6	7	Fruit	76 ± 26.5	4	Whole grains	82 ± 25.3	7	Chicken	91 ± 14	8	Fast foods	137 ± 35	7	Fruits	119 ± 30	6
	Confectionary	47 ± 10.3	6	Beef	73 ± 39	4	Added fats	72 ± 12	6	Potato	89 ± 13	7	Chicken	91 ± 34	4	Added fats	91 ± 10	5
NRF ^d^ (mean ± SD)	425.8 * ± 17.8			**400 ± 24.3**			**488 ± 30.2**			**485 ± 21.3**			**409.4 ± 36.9**			**404.9 ± 19.8**		

^a^ Weighted sample size; ^b^ values are mean (g) ± standard error of the mean (SEM); ^c^ percentage of contribution of the food group; ^d^ nutrient-rich food; * significant values are bolded (*p* < 0.05).

**Table 3:** Dietary patterns of Indigenous women in 2004 and 2015.

	CCHS 2004												CCHS 2015					
	Women												Women					
**Dietary pattern**	Soups Wgt *N* ^a^ = 27,959			Fruits Wgt *N* = 2,983,971			Mixed Wgt *N* = 302,010			Vegetables Wgt *N* = 25,741			Mixed Wgt *N* = 676,074			Healthy-like Wgt *N* = 1,245,903		
**Top five foods**	Food name	Mean ^b^ ± SD (g)	% ^c^	Food name	Mean ± SD (g)	%	Food name	Mean ± SD (g)	%	Food name	Mean ± SD (g)	%	Food name	Mean ± SD (g)	%	Food name	Mean ± SD (g)	%
	1. Soups	358 ± 28	36	Fruits	368 ± 37	34	Vegetables	83 ± 4.9	14	Vegetables	582 ± 51	44	Vegetables	134 ± 9.8	13	Soups	412 ± 45.2	25
	2. Vegetables	78 ± 11	8	Vegetables	97 ± 16	9	Added fats	49 ± 4.2	8	Pasta	105 ± 30	8	Fast foods	117 ± 9.6	11	Fruits	264 ± 38.6	16
	3. Beef	54 ± 16	5	Confectionery	63 ± 25	6	Potato	47 ± 5.1	8	Fruits	99 ± 49	7	Fruits	104 ± 8.8	10	Vegetables	250 ± 39	15
	4. Pasta	47 ± 21	5	Whole grains	51 ± 12	5	Beef	42 ± 4.4	7	Added fats	72 ± 13	5	Whole grains	73 ± 6.3	7	Whole grains	87 ± 12	5
	5. Potato	46 ± 16	5	Added fats	49 ± 6.2	5	Whole grains	34 ± 4	6	Pork	43 ± 14	3	Chicken	71 ± 10	7	Sweet baked goods	76 ± 19	5
NRF ^d^ (mean ± SD)	407 * ± 26			**526.4 ± 28.7**			**455 ± 16.8**			**568.7 ± 42.7**			**481.7 ± 10.9**			**568.4 ± 37.3**		

^a^ Weighted sample size; ^b^ values are mean (g) ± standard error of the mean (SEM); ^c^ percentage of contribution of the food group; ^d^ nutrient-rich food; * significant values are bolded (*p* < 0.05).

**Table 4:** Dietary patterns of Indigenous children in 2004 and 2015.

		CCHS 2004									CCHS 2015					
		Children									Children					
**Dietary pattern**		Soups Wgt *N* ^a^ = 16,287			High fat-High sugar Wgt *N* = 159,354			Mixed Wgt *N* = 27,695			Mixed Wgt *N* = 120,825			Unhealthy Wgt *N* = 39,517		
**Top five foods**		Food name	Mean ^b^ ± SD (g)	% ^c^	Food name	Mean ± SD (g)	%	Food name	Mean ± SD (g)	%	Food name	Mean ± SD (g)	%	Food name	Mean ± SD (g)	%
		1. Soups	406 ± 33	37	Vegetables	66 ± 5.5	11	Fruits	235 ± 20	20	Fruits	161 ± 15	15	Fast foods	411 ± 28	29
		2. Vegetables	89 ± 19	8	Whole grains	43 ± 4.7	7	Confectionery	167 ± 26	14	Vegetables	102 ± 8.3	9	Whole grains	133 ± 16	9
		3. Fruits	79 ± 17	7	Added Fats	42 ± 3	7	Pasta	133 ± 24	11	Sweet baked goods	72 ± 6.7	7	Fruits	93 ± 15	7
		4. Pasta	48 ± 15	4	Confectionery	36 ± 3	6	Vegetables	100 ± 18	9	Whole grains	66 ± 5.9	6	Confectionery	82 ± 14	6
		5. Confectionery	49 ± 11	4	Fast foods	33 ± 3	5	Whole grains	60 ± 12	5	Soups	64 ± 11	6	Sweet baked goods	70 ± 12.7	5
NRF ^d^ (mean ± SD)		425.5 ± 18.6			457.5 ± 12			470.9 ± 36			510.4 ± 9.3 *			**427.8 ± 13.5**		
Age (mean ± SD)		9.5 ± 1			11 ± 0.5			10 ± 0.7			**9.9 ± 0.3**			**11.8 ± 0.6**		
Weight (mean ± SD)		41.4 ± 6.7			48 ± 3.1			51 ± 3.6			**47 ± 2.7**			**57.7 ± 4.4**		
Gender (% women)		44.			59.4			69.3			52			33		
BMI (mean ± SD)		0.8 ± 0.3			0.87 ± 0.1			1.1 ± 0.1			0.9 ± 0.1			1.1 ± 0.3		
Income (%)	Low	71.7			76.3			72.1			34			37		
	High	28.2			23.6			27.8			66			63		
Education (%)	Below = bachelor’s level	96			91			95			83			83		
	Bachelor’s level and above	3.9			8.8			4.8			17			17		
Activity (%)	Active	**100**			**3.1**			**0.5**			**49**			**27**		
	Inactive	**0**			**96.8**			**99.4**			**51**			**73**		
Food security (%)	Food insecure	43.2			52			47			42			44		
	Food secure	56.7			47.9			52.7			58			56		

^a^ Weighted sample size; ^b^ values are mean (g) ± standard error of the mean (SEM); ^c^ percentage of contribution of the food group; ^d^ nutrient-rich food; * significant values are bolded (*p* < 0.05).

**Table 5:** Factors associated with chronic diseases among Indigenous adults.

Variables	CCHS 2004	CCHS 2015
	Adjusted Odds Ratio ^1^ (95% CI)	Adjusted Odds Ratio (95% CI)
Gender		
Men	1 ^#^	1
Women	3.2 (0.8–12.3)	1.2 (0.45–3.3)
Age Groups		
≥30 and <45 years	1	1
≥45 and >65 years	3.3 (0.6–4.7)	5.8 (1.6–6.5) *
≥65 years	5.2 (0.6–6)	**6.9 (1.6–8)**
Education		
No university degree	1	1
A university degree	0.18 (0.01–3)	0.31 (0.06–1.6)
Income		
Low income	1	1
High income	**0.14 (0.04–0.5)**	0.6 (0.2–1.8)
Smoking Status		
Non-smoker	1	1
Smoker	0.35 (0.1–1.1)	1.1 (0.3–1.4)
Physical Activity		
Active	1	1
Inactive	1.5 (0.5–4.5)	1.2 (0.4–3.2)
NRF ^2^		
1st tertile	1	1
2nd tertile	0.3 (0.06–2.1)	0.4 (0.1–1.4)
3rd tertile	0.3 (0.05–2.1)	0.6 (0.1–2.8)
Dietary Patterns	Soups: 1	Mixed: 1
	Fruit/Veg/Pasta: 0.4 (0.03–6.1)	Unhealthy: 2.7 (0.6–4)
	Mixed: 0.8 (0.1–4.9)	
BMI ^3^		
Normal	1	1
Overweight	0.3 (0.09–1.6)	1.4 (0.3–5.4)
Obese	0.8 (0.16–4.7)	3 (0.8–5)

^1^ The OR for each variable was adjusted for every other variable shown in the table. ^2^ Nutrient-rich food (NRF); the reference group is the 1st tertile. ^3^ Body mass index (BMI) (kg/m^2^) for adults. * Significant values are bolded (*p* < 0.05). ^#^ “1” presents the reference group for each variable.

## Data Availability

The data is unavailable due to the privacy and regulations.

## References

[1] World Health Organization (2018). Noncommunicable Diseases Country Profiles. https://apps.who.int/iris/handle/10665/274512.

[2] Hahmann T., Kumar M. (2022). Unmet Health Care Needs during the Pandemic and Resulting Impacts among First Nations People Living Off Reserve, Métis and Inuit. StatCan COVID-19: Data to Insights for a Better Canada. https://www150.statcan.gc.ca/n1/pub/45-28-0001/2022001/article/00008-eng.htm.

[3] Pollex R.L., Zinman B.H.A., Harris S.B., Hegele R.A. (2006). Clinical and Genetic Associations with Hypertriglyceridemic Waist in a Canadian Aboriginal Population. Int. J. Obes..

[4] Weir G.C., Bonner-Weir S. (2004). Five stages of evolving beta-cell dysfunction during progression to diabetes. Diabetes.

[5] Kant A. (2004). Dietary patterns and health outcomes. J. Am. Diet. Assoc..

[6] Truth and Reconciliation Commission of Canada: Calls to Action. https://www2.gov.bc.ca/assets/gov/british-columbians-our-governments/indigenous-people/aboriginal-peoples-documents/calls_to_action_english2.pdf.

[7] Guyot M., Dickson C., Paci C., Furgal C., Chan H.M. (2006). Local observations of climate change and impacts on traditional food security in two northern Aboriginal communities. Int. J. Circumpolar Health.

[8] Sheikh N., Egeland G.M., Johnson-Down L., Kuhnlein H.V. (2011). Changing dietary patterns and body mass index over time in Canadian Inuit communities. Int. J. Circumpolar Health.

[9] Gittelsohn J., Wolever T.M., Harris S.B., Harris-Giraldo R., Hanley A.J., Zinman B. (1998). Specific patterns of food consumption and preparation are associated with diabetes and obesity in a Native Canadian community. J. Nutr..

[10] Carter P., Gray L.J., Troughton J., Khunti K., Davies M.J. (2010). Fruit and vegetable intake and incidence of type 2 diabetes mellitus: Systematic review and meta-analysis. BMJ.

[11] Power E. (2008). Conceptualizing food security for Aboriginal people in Canada. Can. J. Public Health.

[12] Shafiee M., Keshavarz P., Lane G., Pahwa P., Szafron M., Jennings D., Vatanparast H. (2022). Food Security Status of Indigenous Peoples in Canada According to the 4 Pillars of Food Security: A Scoping Review. Adv. Nutr..

[13] Boult D. (2004). Hunger in the Arctic: Food (In) Security in Inuit Communities a Discussion Paper. https://ruor.uottawa.ca/bitstream/10393/30217/1/2004_Inuit_Food_Security.pdf.

[14] McAuley C.K.L. (2011). Impacts of traditional food consumption advisories: Compliance, changes in diet and loss of confidence in traditional foods. Environ. Health.

[15] Ellulu M., Abed Y., Rahmat A., Ranneh Y., Ali F. (2014). Epidemiology of obesity in developing countries: Challenges and prevention. Glob. Epidemic Obes..

[16] Foulds H.J., Bredin S.S., Warburton D.E. (2012). The relationship between hypertension and obesity across different ethnicities. J. Hypertens..

[17] Reedy J., Krebs-Smith S.M., Miller P.E., Liese A.D., Kahle L.L., Park Y., Subar A.F. (2014). Higher diet quality is associated with decreased risk of all-cause, cardiovascular disease, and cancer mortality among older adults. J. Nutr..

[18] McGuire S. (2016). Scientific report of the 2015 dietary guidelines advisory committee. Washington, dc: Us departments of agriculture and health and human services 2015. Adv. Nutr..

[19] Goldman N., Weinstein M., Cornman J., Singer B., Seeman T., Goldman N., Chang M.C. (2004). Sex differentials in biological risk factors for chronic disease: Estimates from population-based surveys. J. Women’s Health.

[20] Misra R., Lager J. (2009). Ethnic and gender differences in psychosocial factors, glycemic control, and quality of life among adult type 2 diabetic patients. J. Diabetes Complicat..

[21] Guggenbuhl P. (2009). Osteoporosis in males and females: Is there really a difference?. Joint Bone Spine.

[22] Diaz H., Marshak H.H., Montgomery S., Rea B., Backman D. (2009). Acculturation and gender: Influence on healthy dietary outcomes for Latino adolescents in California. J. Nutr. Educ. Behav..

[23] Health Canada (2004). Canadian Community Health Survey, Cycle 2.2, Nutrition (2004): A Guide to Accessing and Interpreting the Data. Ottawa, ON: Health Canada. http://www.hc-sc.gc.ca/fn-an/surveill/nutrition/commun/cchs_guide_escc-eng.php.

[24] Canadian Community Health Survey (CCHS) Reference Guide to Understanding and Using Data 2015 Canadian Community Health Survey—Nutrition. Government of Canada. http://www23.statcan.gc.ca/imdbbmdi/pub/document/5049_D23_T9_V1-eng.pdf.

[25] US Department of Agriculture & Agricultural Research Service (2009). USDA Automated Multiple-Pass Method. http://www.ars.usda.gov/Services/docs.htm?docid=7710.

[26] Moshfegh A.J., Borrud L., Perloff B., LaComb R. (1999). Improved method for the 24-hour dietary recall for use in national surveys. FASEB J..

[27] Maillot M., Vieux F., Delaere F., Lluch A., Darmon N. (2017). Dietary changes needed to reach nutritional adequacy without increasing diet cost according to income: An analysis among French adults. PLoS ONE.

[28] Barr S.I., Vatanparast H., Smith J. (2020). Breakfast in Canada, revalence of Consumption, Contribution to Nutrient and Food Group Intakes, and Variability across Tertiles of Daily Diet Quality. A Study from the International Breakfast Research Initiative. Nutrients.

[29] World Health Organization (WHO) WHO AnthroPlus Software. https://www.who.int/growthref/tools/en/.

[30] Makles A. (2012). Stata tip 110: How to get the optimal k-means cluster solution. Stata J..

[31] Garriguet D. (2007). Canadians’ eating habits. Health Rep..

[32] Garriguet D. (2008). Obesity and the eating habits of the Aboriginal population. Health Rep..

[33] Johnson-Down L., Labonte M.E., Martin I.D., Tsuji L.J., Nieboer E., Dewailly E., Egeland G., Lucas M. (2015). Quality of diet is associated with insulin resistance in the Cree (Eeyouch) indigenous population of northern Quebec. Nutr. Metab. Cardiovasc. Dis..

[34] Sheehy T., Kolahdooz F., Roache C., Sharma S. (2015). Traditional food consumption is associated with better diet quality and adequacy among Inuit adults in Nunavut, Canada. Int. J. Food Sci. Nutr..

[35] Kuhnlein H.V., Receveur O., Soueida R., Egeland G.M. (2004). Arctic indigenous peoples experience the nutrition transition with changing dietary patterns and obesity. J. Nutr..

[36] Chan H.M., Fediuk K., Batal M., Sadik T., Tikhonov C., Ing A., Barwin L. (2019). First Nations Food, Nutrition and Environment Study (FNFNES): Results from Quebec 2016.

[37] Chan L. (2012). First Nations Food, Nutrition and Environment Study (FNFNES).

[38] Chan L. (2017). First Nations Food, Nutrition and Environment Study (FNFNES): Results from the Atlantic 2014.

[39] Setiono F.J., Jock B., Trude A., Wensel C.R., Poirier L., Pardilla M., Gittelsohn J. (2019). Associations between Food Consumption Patterns and Chronic Diseases and Self-Reported Morbidities in 6 American Indian Communities. Curr. Dev. Nutr..

[40] Ng C., Corey P.N., Young T.K. (2011). Socio-economic patterns of obesity among aboriginal and non-Aboriginal Canadians. Can. J. Public Health.

[41] Sarkar D., Walker-Swaney J., Shetty K. (2020). Food diversity and indigenous food systems to combat diet-linked chronic diseases. Curr. Dev. Nutr..

[42] Gracey M. (2000). Historical, cultural, political, and social influences on dietary patterns and nutrition in Australian Aboriginal children. Am. J. Clin. Nutr..

[43] Langlois K.A., Findlay L.C., Kohen D.E. (2013). Dietary habits of Aboriginal children. Health Rep..

[44] Foulds H.J., Bredin S.S., Warburton D.E. (2012). An evaluation of the physical activity and health status of British Columbian Aboriginal populations. Appl. Physiol. Nutr. Metab..

[45] Newby P.K., Tucker K.L. (2004). Empirically derived eating patterns using factor or cluster analysis: A review. Nutr. Rev..

